# Endophytic Microbiome Responses to Sulfur Availability in *Beta* *vulgaris* (L.)

**DOI:** 10.3390/ijms22137184

**Published:** 2021-07-02

**Authors:** Giovanni Bertoldo, Maria Cristina Della Lucia, Andrea Squartini, Giuseppe Concheri, Chiara Broccanello, Alessandro Romano, Samathmika Ravi, Massimo Cagnin, Andrea Baglieri, Piergiorgio Stevanato

**Affiliations:** 1 Department of Agronomy, Food, Natural Resources, Animals and Environment-DAFNAE, Agripolis, University of Padova, 35020 Legnaro, Italy; giovanni.bertoldo@phd.unipd.it (G.B.); mariacristina.dellalucia@phd.unipd.it (M.C.D.L.); squart@unipd.it (A.S.); giuseppe.concheri@unipd.it (G.C.); chiarabr87@yahoo.it (C.B.); samathmikaravi@gmail.com (S.R.); massimo.cagnin@unipd.it (M.C.); 2 Plant Protection and Certification Centre, Council for Agricultural Research and Economics, 36045 Lonigo, Italy; alessandro.romano@crea.gov.it; 3 Department of Agriculture, Food and Environment, University of Catania, 95123 Catania, Italy; abaglie@unict.it

**Keywords:** Sulfur, sugar beet, endophytic community, Next Generation Sequencing (NGS)

## Abstract

Sulfur is an essential plant macronutrient, and its adequate supply allows an efficient root storage and sugar extractability in sugar beets (*Beta* *vulgaris* L.). In this study, we investigated the effect of changes in sulfur availability on the endophytic community structure of sugar beets. Plants were hydroponically grown in a complete nutrient solution (S-supplied), a nutrient solution without MgSO_4_ (S-deprived), and a nutrient solution without MgSO_4_ for six days and resupplied with 100 μM MgSO_4_ for 48 h (S-resupplied). The sulfur status was monitored by inductively coupled plasma ICP–OES, and combustion analysis together with the evaluation of microRNA395 as a biomarker for sulfate status. Metabarcoding of the bacterial 16S rRNA gene was carried out in order to determine leaf endophytic community structure. The Shannon diversity index significantly differed (*p* < 0.05) between sulfate-supplied and sulfate-deprived seedlings. Validation by Real-Time PCR showed a significant increase (*p* < 0.05) of *Burkholderia* spp. in sulfate-deprived plants as compared to sulfate-supplied ones. The study sheds new light on the effects of nutrient deficiency on the microbiome of sugar beet plants.

## 1. Introduction

Abiotic stresses, such as a nutrient deficiency, can shape the presence and number of plant growth-promoting bacteria recruited from the environment. In sugar beets (*Beta vulgaris* L.), sulfur deficiency affects the sugar yield and technological quality of roots [1]. Sulfur is an essential plant nutrient, taken in by roots and metabolized in leaves. It is required to synthesize key amino acids such as methionine and cysteine, which are needed to produce functional and structural proteins [2]. In sugar beets, low S concentration leads to the accumulation of alfa-amino N, resulting in less root storage capacity and lower sugar extractability. Studies have revealed that, in S-deficient plant tissues, non-S containing amino acids such as glutamine and asparagine accumulate, in addition to non-protein compounds such as amides [3]. Moreover, sulfate plays an important role in crop health and stress tolerance. As an example, glutathione (one of the forms of organic S) can act as a messenger, carrying information on fungal attacks [4,5].

Research on plant microbiomes has been attracting huge interest and investments to clarify the role of plant-associated microorganisms. The plant microbiome is an essential indicator of a plant’s physiological condition, and the effect of biotic and abiotic stresses can alter this fragile balance and lead to pathological conditions [6]. Bacteria, fungi, and viruses, which constitute the plant microbiome, can interact with each other and with the plant itself in ways that may result in stress-coping adaptation, enhancing nutrient uptake, plant growth, and morphogenesis [7]. In this scenario, bacteria communicate with plants through the release of phytohormones, small molecules, and volatile substances. Plants, on their side, release a variety of molecules such as vitamins, organic acids, and sugars used by bacteria as nutrients or signals [8]. The composition of bacterial communities differs among plant species and tissues [9]. Also, studies have revealed that microbiome changes are detectable according to plant developmental stage, seasonality, nutritional status, and the presence of stress [10]. The plant’s aerial part (or the phyllosphere) is an extreme habitat directly exposed to many abiotic stresses including UV, low water and nutrient availability, temperature, salinity, and pressure [11]. This environment selects only microorganisms able to grow and survive under all environmental conditions and to acquire specific adaptation mechanisms. Therefore, differences in microbiome composition can be detectable on a leaf’s surface [12]. The epiphytic and endophytic microbial communities of the phyllosphere play a crucial role in processes related to plant development such as the biological control of diseases and the synthesis of phytohormones such as auxin, cytokinin, and gibberellin, which affect growth through modulating endogenous hormone levels in association with their plant-derived counterparts [13,14].

Generally, endophytic bacteria are non-pathogenic organisms and do not cause visible damage or morphological changes in their hosts. Several studies have highlighted the effectiveness of endophytic bacteria in protecting plants from abiotic stresses such as drought, salinity, and low temperature and in mediating plant defense response to pathogenic attacks [15]. To describe the plant’s microbiome, the advent of sequencing technologies has revolutionized the method of detecting microbial species, also expanding it to those species that are non-culturable and hence unsuitable to be studied with traditional methods [16]. The 16S rRNA gene, composed of nine hypervariable regions interspaced with highly conserved elements, is commonly sequenced to reveal the specific taxonomy of the bacterial species upon database alignments [17].

In this study, the effect on plant endophytic community structure, following changes in sulfur availability, was investigated. Leaf sulfur status was monitored by inductively coupled plasma ICP–OES, and combustion analysis together with an evaluation of microRNA395 as a biomarker for sulfate status [18]. Then, metabarcoding of bacterial 16S rRNA gene was carried out to determine leaf endophytic community structure in response to changes in sulfate availability. From the metagenomic sequencing information, we designed specific primers to validate and quantify the presence of the detected bacteria with Real-Time PCR. The specific aim was to detect plant-growth-promoting and/or specific physiological condition-indicator bacteria in sugar beet seedlings following a sulfur deficiency condition.

## 2. Results

**Figure 1:**
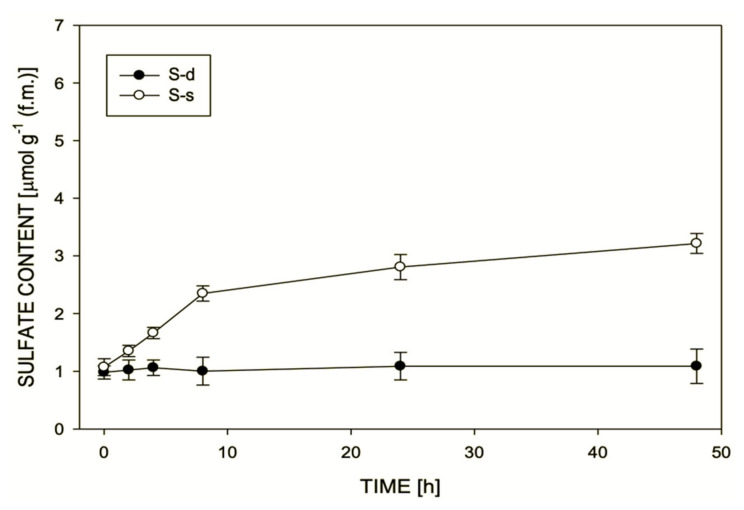
Sulfate content of S-deprived (S-d) and S-supplied (S-s) plants were obtained after 2, 4, 8, 24, and 48 h of treatment. The graph shows the means of replicates (12 observations each time) and error bars represent the standard error of the means.

To verify the effectiveness of nutritional treatments, the sulfur status of S-deprived and S-resupplied plants were firstly evaluated with the optical emission spectroscopy with inductively coupled plasma (ICP–OES). The sulfur content was also monitored by combustion analysis, and results were significantly correlated (*p* < 0.01) with them obtained by ICP–OES. The sulfate content of plants grown at 100 μM sulfate was significantly higher (*p* < 0.01) than a plant grown in a Hoagland solution without MgSO_4_, as represented in Figure 1.

**Figure 2:**
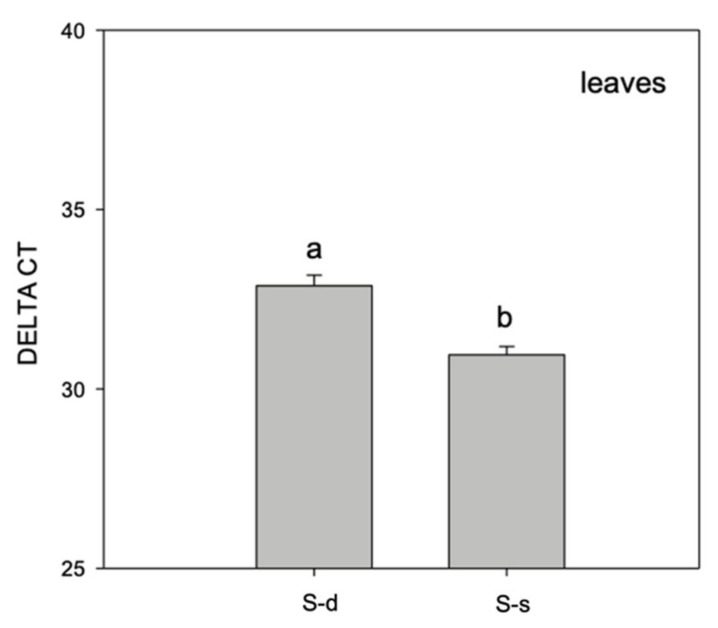
Bar plot showing the expression of miR395 in S-deprived (S-d) and S-supplied (S-s) plants for 48 h. Means of replicates and standard error is reported. The experiment has been performed three times to validate the results.

At the molecular level, we proceeded to test the expression of the marker miR395. The presence of this marker was reduced in plants grown with high S availability as shown in Figure 2.

**Figure 3:**
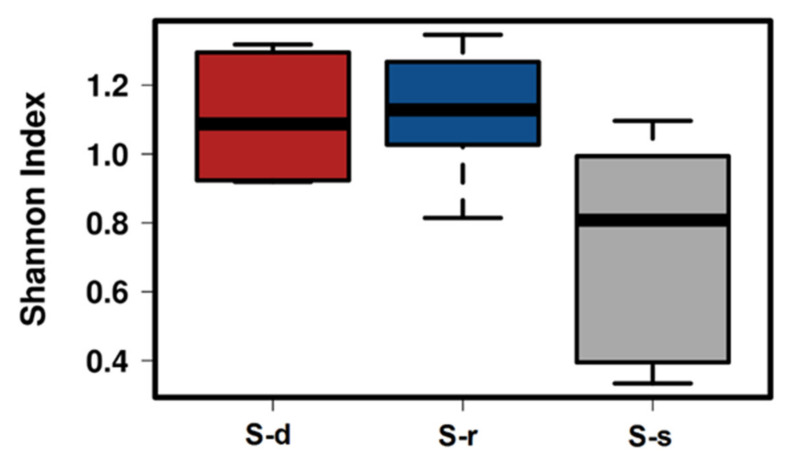
Boxplots of Shannon Index reflecting the diversity of bacterial microbiomes in S-deprived (S-d), S-resupplied (S-r), and S-supplied (S-s) plants. Boxes represent the interquartile range between the first and third quartile. The horizontal line inside the box defines the median (ANOVA; α < 0.05).

A total of 2,395,918 sequences were obtained for 18 samples and, among these, 1,845,486 were considered valid. The average length of the obtained sequences was 241 nucleotides, with a median and mode of, respectively, 260 and 216 nucleotides. At 97% identity cut-off, 149 OTUs, classified into 34 families, 41 genera, and 74 species, were detected using the Greengenes database version 13.5 (public database available at https://greengenes.secondgenome.com/?prefix=downloads/greengenes_database/) and the curated MicroSEQ^®^ 16S Reference Library v2013.1 (Thermo Fisher Scientific Inc., Waltham, MA, USA) on the Torrent Suite Software 5.12 (Thermo Fisher Scientific Inc., Waltham, MA, USA). Plant bacterial diversity significantly differed among treatments with a *p*-value of 0.024 calculated using the Shannon index (Figure 3).

**Figure 4:**
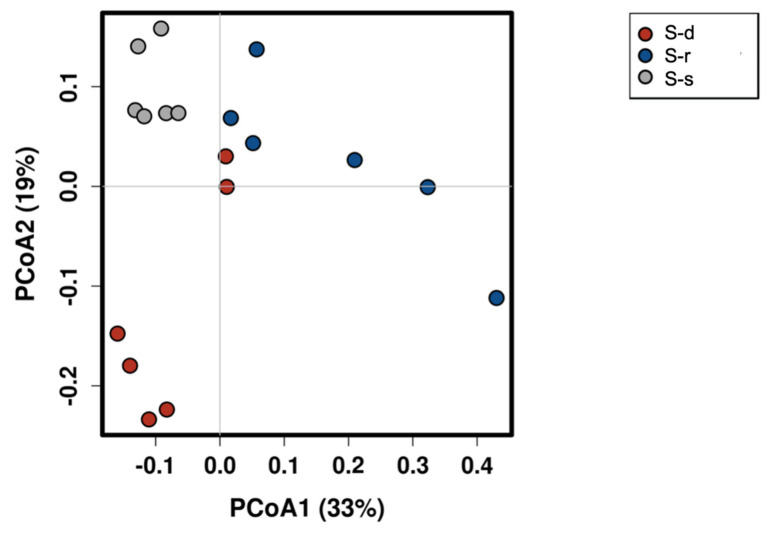
Principal Coordinates Analysis (PCoA) plots of Bray-Curtis distances between S-deprived (S-d), S-resupplied (S-r), and S-supplied (S-s) plants with the percentage variance explained by the principal coordinates.

A Bray–Curtis distance genus-level PCoA analysis was plotted to visualize clustering of samples (Figure 4). The first two principal coordinate axes accounted for 33% and 19% of the variation. Particularly, the S-supplied plants can be clearly distinguished from the S-deprived and S-resupplied plants that clustered independently.

**Figure 5:**
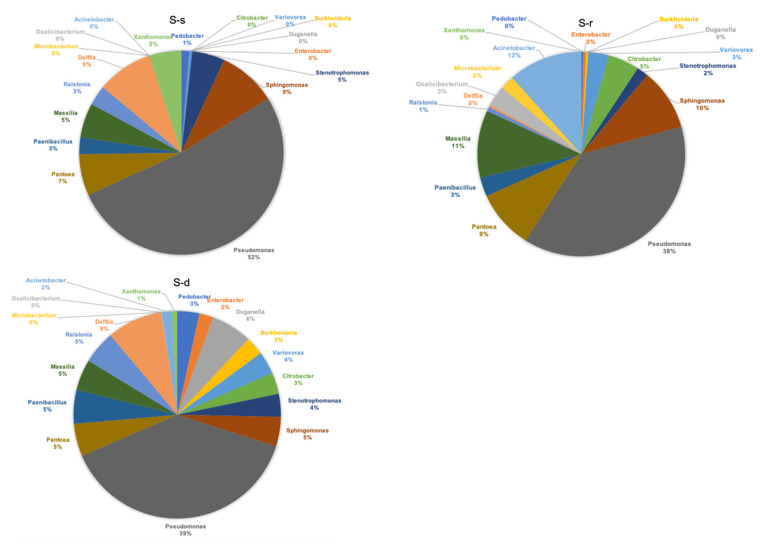
Pie charts showing bacteria genera abundance expressed as a percentage over the total detected in S-supplied (S-s), S-resupplied (S-r), and S-deprived (S-d) plants.

The bacterial community composition in the leaves was represented by pie charts, based on the relative bacterial genera abundance (Figure 5).

**Figure 6:**
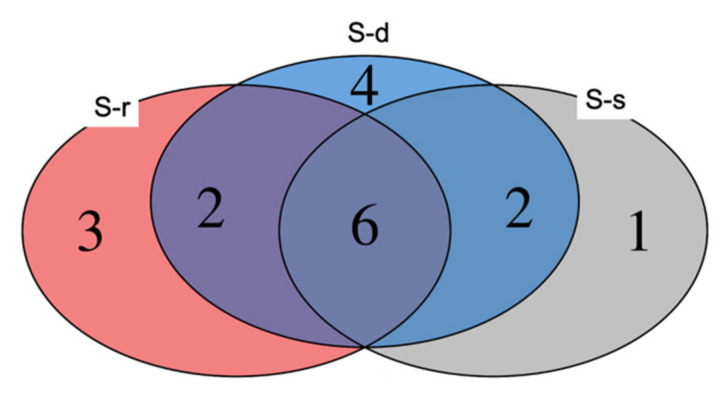
Venn diagram showing the number of shared and exclusive OTUs. Areas 1, 3, and 4 represent OTUs found exclusively in S-supplied (S-s), S-resupplied (S-r,), and S-deprived (S-d) plants, respectively. Overlapping area 6 represents the OTUs in common between all treatments.

*Pseudomonas* was the most abundant genus across the three treatments reaching 39% and 38%, respectively, for S-deprived and S-resupplied samples and 52% for S-supplied plants. In addition to *Pseudomonas*, five other genera were found in all treatments (Figure 6). These were: *Stenotrophomonas*, *Sphingomonas*, *Pantoea*, *Paenibacillus*, and *Massilia*. Among the other bacteria, four were found only on S-deprived plants: *Pedobacter*, *Enterobacter*, *Duganella*, and *Burkholderia*. The genera *Oxalicibacterium*, *Microbacterium*, *Acinetobacter* were found only on S-resupplied plants (Figure 6). The only genus found on S-supplied plants was *Xanthomonas* (Figure 6).

**Figure 7:**
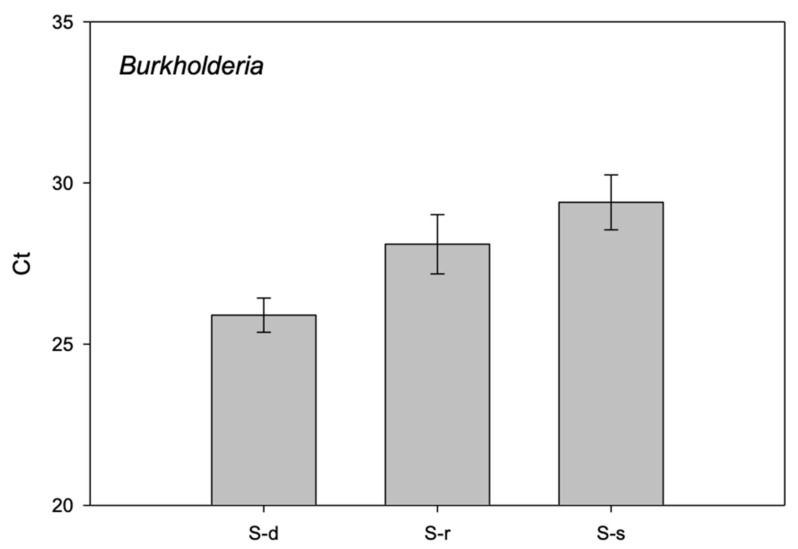
Relative abundance by qPCR of *Burkholderia* genera in S-deprived (S-d), S-supplied (S-s), and S-resupplied (S-r) plants. Threshold cycle means and corresponding standard errors are reported (the lower the values, the higher the number of target gene copies within plant tissues). Vertical bars indicate standard errors.

Subsequently, we validated by qPCR the sequencing results using specific primer pairs to amplify and quantify the presence of bacteria. The qPCR technique was performed on the stored DNA not used for library preparation and coming from plant samples of the first, second and third growing cycle. The results obtained confirmed the presence of *Pseudomonas* genus in all treatments, with no significant difference among them (*p* = 0.5767) (Figure 7). The most significant differences across treatments were found for *Burkholderia*, which showed an average Ct of 25.9 on S-deprived samples, 28.1 on S-resupplied samples, and 29.4 on S-supplied samples (Figure 7).

The other bacteria that were only found on S-deprived, S-resupplied, and S-supplied plants, were not confirmed by Real-Time PCR analysis on the other two growing cycles, since the differences between groups were not significant (data not shown).

## 3. Discussion

Environmental conditions and biotic and abiotic stresses can shape a plant’s microbiome. In our study, germination took place in a growth chamber with distilled water-moistened filter paper, and seedlings were then transferred and grown in a hydroponic solution. These conditions not only allow the presence and detection of seed and plant endophytes but also bacteria naturally present in the water and surrounding environment, which can be considered as contaminants hardly avoidable during DNA isolation steps. However, we focused our attention on the microorganisms in common between treatments as constituents of the seed microbiome inherited by vertical transmission, and on the microorganisms that are specifically present in each treatment to identify those most related to overcoming nutritional stress. Since sulfur metabolism is known to take place in the leaf, we described the microbiome in the outer and inner parts of this tissue, the phyllosphere and endosphere, respectively.

To assess the effective plant sulfur nutritional status, we adopted two different techniques. The optical emission spectroscopy with inductively coupled plasma (ICP–OES) has widely been used to monitor the overall nutritional status of plant tissue, detect micronutrient quantity, and evaluate the presence of heavy metals contamination [19]. In our study, this technique was employed as a first step to assess the real sulfur presence or absence inside leaves. Moreover, at the molecular level, the Real-Time quantitative RT–PCR quantified the expression of a specific microRNA called miR395. This marker has been previously adopted to analyze the sulfate starvation in *Beta vulgaris*, *Arabidopsis*, and sorghum [20,21,22]. Here, the shortage of sulfate was connected to an increased expression of miR395, following the same trend observed in the previous paper. Once we detected the effective sulfur nutritional status, we proceed to analyze its relationship with leaf endophytes.

Plant bacteria diversity analysis with Shannon index revealed a significant bacteria diversity of sulfur-supplied plants compared to the sulfur-deprived and sulfur-resupplied plants. Specifically, 48 h of treatments with MgSO_4_ seems to be not enough to restore the presence of bacteria in stressed plants. The same result has also been confirmed by PCoA, where S-deprived and S-resupplied plants clustered together. However, this speculation should be confirmed by a more dedicated experiment, since until now there is no evidence in the available literature.

In our study, the most abundant detected endophyte was *Pseudomonas*. This bacterium is commonly reported in seeds of very different plant species, and also usually present in the phyllosphere, with a very important ecological function. *Pseudomonas* is known as a plant-growth-promoting bacterium capable of secreting 1-aminocyclopropane-1-carboxylate (ACC) deaminase to enhance plant growth. As a constituent of the phyllosphere, *Pseudomonas* is reported to limit the loss of water and damage due to UV radiation [23]. Moreover, a high diversity of *Pseudomonas* species consortia is known to be positively correlated with the accumulation of plant biomass and the assimilation of nutrients [24]. Among the constituents of the core microbiome, we detected *Sphingomonas*, *Pantoea*, *Paenibacillus*, *Massilia*, and *Stenotrophomonas*. *Sphingomonas* is a gram-negative bacterium with an essential role in environmental remediation, capable of accumulating intracellular Zn^2+^ and reducing the uptake of Cd^2+^ by plant roots. This genus can bind the heavy metal and enhance the expression of cysteine-rich metallothionein proteins [25]. *Sphingomonas* also have an important role in counteracting biotic and abiotic stresses, such as the mitigation of salinity stress [26]. A similar role was reported by Grover et al. [27] for *Pantoea* and *Paenibacillus* as PGPB providing tolerance to different abiotic stress environments in tomato, pepper, canola, bean, and lettuce. Moreover, the genus *Stenotrophomonas* has been reported to be highly adaptable to nutrient-limited environments colonizing and surviving on the plant surface. Also, this genus contributes to enhancing growth conditions for bacteria by increasing water permeability [28].

Analyzing the microbiome composition of S-deprived, S-resupplied, and S-supplied plants, we revealed the presence of very distinct genera for each treatment. These genera were validated with Real-Time PCR on the same sample used for sequencing analysis and on plants from additional growing cycles. The presence of *Pseudomonas* was confirmed on all plants among replicates without any significant difference between treatments. *Pedobacter*, *Enterobacter*, *Duganella*, *Microbacterium*, *Oxalicibacter*, *Acinetobacter*, and *Xanthomonas* showed a variable presence, but not significant, in all samples and among replicates. However, the Real-Time PCR results confirmed a significant difference of *Burkholderia* among the three treatments. Specifically, the *Burkholderia* genus was abundant in S-deprived plants. This genus includes versatile bacteria that survive in many ecological niches, from soil to animals and humans [29]. The type of interaction with the plant host can be pathogenic, symbiotic, or both. *Burkholderia* species are mainly known as the most potent plant-growth-promoting rhizobacteria, with colonization reported for at least 30 plant species [30]. The molecular mechanisms underlying the interaction between these microorganisms and the host plant are still unknown. However, *Burkholderia* species are recognized for the benefits they bring to agriculture. Among these, the most important are nitrogen fixation, IAA and siderophore production, inorganic phosphate solubilization, and pathogen inhibition [31,32]. This genus is also capable of epiphytic and endophytic colonization of grapevine tissues and organs protecting the plants against heat as well as chilling stress [33]. Similarly, *Burkholderia* increased drought stress resilience of maize [34], induced metabolic changes in *Arabidopsis* under salt tolerance [35] and improved the growth, physiology, and antioxidant activity of *Brassica napus* L. in chromium-contaminated soil [36].

This evidence let us speculate that a plant under abiotic stress could foster the growth of specific microorganisms in the background of its hosted taxa, particularly boosting those that can protect it and leading to their relative abundance shifts in comparison to others that would not be as efficient in helping to cope with that particular stress factor. This could therefore have happened to our plants subjected to the nutritional stress of sulfur deficiency. Having this result been repeatedly confirmed in three growing cycles, it stands as a confidence–worth observation.

In conclusion, the evidence gathered from our results suggests that: (1) seed-borne bacteria constitute a consistent presence in sugar beet, featuring a large contingent of various taxa; and (2) within the ‘inner spore bank’ offered by these, the plant would respond to nutritional stress as early as at seedling stage by inducing and allowing taxon-specific endophytic growth (this selective enhancement can supposedly be exerted by increased synthesis of defined chemical compounds). Such a strategy is already known to shape microbial communities in the outer rhizosphere. The observed patterns indicate that this mechanism is operative also in the inner tissues and that it preferentially fosters the bacteria that could be needed to respond to a given stress, upshifting them immediately after seed germination. The further investigation focused on the dynamics of endophytic plant growth-promoting bacteria offer several promising developments for future trends in improved agriculture and horticulture.

## 4. Material and Methods

### 4.1. Plant Material

The seeds of sugar beet (*Beta vulgaris* (L.)) used in this study belong to the “Shannon” variety provided by Lion Seeds Ltd. (Maldon, UK). This genotype is a hybrid obtained crossing a multigerm pollinator resistant to rhizomania with a susceptible male-sterile monogerm line.

### 4.2. Growing Conditions

The sugar beet seeds were surface sterilized by dipping them in an ethanol solution (76%) for 5 min. The treatment was repeated three times alternating it with a rinse with distilled water. Seeds were then placed on Petri dishes with distilled water-moistened filter paper and left to germinate for 48 h at a temperature of 25 °C in the dark. Germinated seeds were then transferred into three 500 mL glass pots with Hoagland solution [37]. Thirty seedlings were maintained in a pot for 8 days with a full Hoagland nutrient solution containing MgSO_4_ corresponding to 100 μM sulfate (S-supplied plants). In parallel, other 30 seedlings were maintained for 8 days in a Hoagland solution without MgSO_4_ (S-deprived plants). Further 30 seedlings were initially maintained for 6 days in a Hoagland solution without MgSO_4_ and then transferred into another pot with a full Hoagland nutrient solution containing 100 μM sulfate (S-resupplied plants) and kept in that condition for 48 h. The experiment was repeated three times for validation aims.

### 4.3. Ionomic Analysis

Sulfate content was determined using inductively coupled plasma optical emission spectrometry (ICP–OES, Spectro, Kleve, Germany). Firstly, fresh leaves were ground to powder in liquid nitrogen. Then, sulfate was extracted in 20 cm^3^ of millipore water by incubation at 70 °C for 30 min and centrifuged at 20,000× *g* for 30 min. The supernatant was filtered using a 0.45-μm pore size filter and analyzed both by ICP–OES instrument (Spectro Analytical Instruments, Kleve, Germany), and combustion analysis (Elementar vario MACRO CNS, Elementar Analysensysteme GmbH, Hanau, Germany).

### 4.4. RNA Extraction

Total RNA was extracted from 100 mg of the fresh leaf using the Eurogold TriFastTM kit (EuroClone, Milan, Italy), following the manufacturer’s instructions. RNA quantification was performed with Qubit RNA High Sensitivity Assay Kit (Thermo Fisher Scientific Inc., Waltham, MA, USA).

### 4.5. Real-Time Quantitative RT–PCR

The reverse transcription was achieved using QuantiMir kit (System Biosciences, Mountain View, CA, USA), according to the manufacturer’s instructions, starting from 1 μg of total RNA. Quant Studio 12K Flex Real-Time PCR (Thermo Fisher Scientific Inc., Waltham, MA, USA) was used to detect the expression of miR395 with the following reaction mixture: 5 μL of Power Syber Green PCR master mix (Life Technologies, Carlsbad, CA, USA), 0.1 μL of forward primer, 0.1 μL of reverse primer, 1.4 μL of nuclease-free water, and 1 μL of each sample. The PCR program was set up with an initial denaturation at 95 °C for 10 min followed by 50 cycles at 95 °C for 15 s and 60 °C for 1 min. The primer sequence used to detect miR395 was obtained from [20,21,22,23,24,25,26,27,28,29,30,31,32,33,34,35,36,37,38]. The threshold method was used to analyze miR395 expression and the ΔCt values, calculated based on Hajizadeh et al. [39], involved the use of 18S rRNA as an internal standard.

### 4.6. DNA Extraction

After 8 days since the transfer of germinated seeds into Hoagland solutions, fresh leaves were collected from each pot and processed to extract DNA. For each leaf sample, 50 mg was homogenized with 300 µL of RLT buffer in two ml Eppendorf tubes using a Tissue Lyser (Qiagen, Hilden, Germany) for 5 min at 30 hertz. The tubes were centrifuged at 6000 rpm for 3 min, and the supernatant obtained was processed using Biosprint 96 (Qiagen, Hilden, Germany) to extract DNA. The automatic extraction involved the use of six 96 well S-Block plates set up as follows. One plate filled with 300 µL of sample supernatant, 200 µL of isopropanol, and 20 µL of MagAttract magnetic beads (Qiagen, Hilden, Germany). A second plate with 500 µL of buffer RLT. The third and fourth plates with 500 µL of 96% ethanol. The fifth plate with 500 µL of 0.02% Tween solution and the last plate filled with 100 µL of nuclease-free water. After the extraction process, DNA was quantified with a Qubit fluorimeter (Thermo Fisher Scientific Inc., Waltham, MA, USA) using the Qubit™ DNA HS Assay Kit (Thermo Fisher Scientific Inc., Waltham, MA, USA).

### 4.7. Metabarcoding of Bacterial 16S rRNA Gene Sequencing and Data Analysis

DNA sequencing was performed with the Ion GeneStudio S5 instrument using a 16S Ion Metagenomics Kit (Thermo Fisher Scientific Inc., Waltham, MA, USA) following the manufacturer’s instructions. The kit provides two separate primer tubes to amplify the regions V2, V4, V8, and V3, V6–7, V9, respectively. The amplification program of the 16S hypervariable regions involved an initial step of 10 min at 95 °C followed by 25 cycles at 95 °C for 30 s, 58 °C for 30 s, 72 °C for 20 s and a hold at the stage at 72 °C for 7 min. Pooled amplicons were used for library preparation with an Ion Xpress Plus Fragment Library Kit (Thermo Fisher Scientific Inc., Waltham, MA, USA). Unique barcodes were ligated to each library using an Ion Xpress Barcode Adapter (Thermo Fisher Scientific). A 6-cycle PCR reaction was performed to amplify libraries at 58 °C for 15 s and 70 °C for 1 min, then at 4 °C for up to one hour. PCR reactions were performed in a SimpliAmp Thermal Cycler (Thermo Fisher Scientific Inc., Waltham, MA, USA).

Twenty-five pM of the pooled libraries were used to prepare template-positive Ion Sphere Particles (ISPs) with One-Touch 2 and One-Touch ES system (Thermo Fisher Scientific Inc., Waltham, MA, USA) and subsequently sequenced with Ion GeneStudio S5 on an Ion 520 chip (Ion 520 Chip Kit, Thermo Fisher Scientific Inc., Waltham, MA, USA).

Sequencing data were analyzed with Torrent Suite software (Thermo Fisher Scientific Inc., Waltham, MA, USA). Ion Reporter cloud software (version 5.16, Thermo Fisher Scientific Inc., Waltham, MA, USA) was adopted to process 16S metagenomic data. A BaseCaller module filtered out the low-quality sequences, together with barcode assignment, adaptor trimming at 3′ end, and base calling. OTU clustering was done against the curated Greengenes database v.13.8 and curated MicroSEQ 16S Reference Library v2013.1, using a read abundance threshold of 10 and 97% sequence similarity. Shannon index was calculated for the three groups to compare the bacterial diversity and PCoA based on the bacterial abundances was also used to determine the overall similarity between groups using the Calypso tool [40].

### 4.8. Primer Design and Real-Time PCR

The 16S DNA sequences of the bacteria, which characterize the three treatments, were used to design forward and reverse primers using the Primer Express V3.0 software (Thermo Fisher Scientific Inc., Waltham, MA, USA). The primer sequences are reported in Table 1.

A QuantStudio 12K Flex unit (Thermo Fisher Scientific Inc., Waltham, MA, USA) was used to perform Real-Time PCR with the following mix: 5 μL of Sybr Green Real-Time PCR Master Mix (Life Technologies, Carlsbad, CA, USA), 0.1 μL of forward primer, 0.1 μL of reverse primer, and 1.4 μL of nuclease-free water. One μL of the sample was analyzed for each well. The cycling program consisted of 10 min of pre-incubation at 95 °C, 50 cycles of 15 s at 95 °C, and 1 min at 60 °C.

## Figures and Tables

**Table 1:** Forward and reverse primer sequences of bacteria species characterizing the three treatments designed by Primer Express V3.0 software.

Name	Primer Forward 5′,3′	Primer Reverse 5′,3′
*Pseudomonas*	GCGCGTAGGTGGCTTGATAA	GGATGCAGTTCCCAGGTTGA
*Pedobacter*	CTGGTCCGGTGTCTCAGTAC	GGGGATCTGAGAGGATGACC
*Enterobacter*	CATGGGAGTGGGTTGCAAAA	TCACAAAAGTGGTAAGCGCC
*Duganella*	GCTACTGATCGATGCCTTGG	GCGGCCGATATCTGATTAGC
*Burkholderia*	CCTCTGCCATACTCTAGCCC	ATGTGAAATCCCCGGGCTTA
*Microbacterium*	GGTACCGTCACTTTCGCTTC	GTGAGGGATGACGGCCTT
*Oxalicibacterium*	GCGCAACCCTTGTCATTAGT	TGTCACCGGCAGTCTCATTA
*Acinetobacter*	GCGAGGAGGAGGCTACTTTA	CGGTGCTTATTCTGCGAGTA
*Xanthomonas*	AAGGTGGGGATGACGTCAAG	TGTGTAGCCCTGGTCGTAAG

## Data Availability

Not applicable.
